# Supplementary material for: Polygenic backgrounds influence phenotypic consequences of variants in cells, individuals, and populations
Source: Cell Genom. 2026 Jan 26;6(2):101131. doi: 10.1016/j.xgen.2025.101131 (PMC12903375; doi:10.1016/j.xgen.2025.101131)
Supplement: Document S1. Supplemental methods — Modelling method details, related to Figures 1 and 2. [file mmc1.pdf]

Cell Genomics, Volume *6*

Supplemental information

Polygenic backgrounds influence phenotypic consequences of variants in cells, individuals, and populations

Madison Chapel, Jessica Dennis, and Carl G. de Boer

Supplementary Information: Polygenic backgrounds influence phenotypic consequences of variants in cells, individuals, and populations

Madison Chapel, Jessica Dennis, Carl G. de Boer

Method Details

Modeling disease risk across polygenic backgrounds

To demonstrate how the effect of a variant changes depending on the genetic background in which it occurs (**Fig. 1**), a range of polygenic scores between –4 and 4 were plotted in $\log_2$ space (log odds ratio; log(OR)) and linear space (odds ratio; OR). A large-effect variant with an effect size of one PGS unit was modeled by shifting the PGS from -2 to -1 for a low-risk background, and from 3 to 4 for a high-risk background (**Fig. 1A,B**).

Disease prevalence across the range of PGS (**Fig. 1C,D**) was calculated by first determining the odds of disease in the reference group based on disease prevalence *(p)* for five different background rates of disease*:*

$$Odds = \frac{1}{1-p}$$

We then determined disease prevalence across the range of corresponding odds ratios (*OR*):

$$p_{OR} = \frac{OR \cdot odds}{1 + (OR \cdot odds)}$$

Impact of polygenicity degree on PGS distributions

To model how variance in the PGS distribution increases as more genes contribute to the disease trait (**Fig. 2**), sample populations were generated for increasing numbers of genes, ranging from 50 to 2000 in increments of 50. For each gene count, 10 replicate populations of 1000 individuals were generated. Allele frequencies for each replicate were generated from a uniform distribution *U*(0.0, 1.0). Additive effect sizes, β, were generated from a normal distribution *N*(mean = 0.0, standard deviation = 0.01). Epistatic effect sizes, γ, which are expected to be much smaller than additive effects[1], were generated from a normal distribution *N*(mean = 0.0, standard deviation = 0.0004). Individual genotypes, χ, were simulated using a binomial distribution with *n*=2 and *p* equal to the generated allele frequencies.

In the additive model, disease liability, *L*, was determined by summing the products of effect sizes and genotypes:

$$L_{add} = \sum_{i=1}^{n} \beta_i \chi_i$$

In the model including epistatic interactions, liability was calculated as:

$$L_{epi} = \sum_{i=1}^{n} \beta_i \chi_i + \sum_{i \leq j}^{n} \gamma_{ij} \chi_i \chi_j$$

Where γ_{ij} is the interaction effect between SNPs *i* and *j*. This model includes only additive effects and additive × additive (i.e., epistatic) interaction effects but omits dominance-related interaction terms[2,3].

Key resources table

REAGENT or RESOURCE	SOURCE	IDENTIFIER
Software and algorithms		
Polygenic background simulations	This paper	https://github.com/de-Boer-Lab/PolygenicBackgroundSims

Supplementary References

1. Zuk, O., Hechter, E., Sunyaev, S.R., and Lander, E.S. (2012). The mystery of missing heritability: Genetic interactions create phantom heritability. Proc. Natl. Acad. Sci. U.S.A. *109*, 1193–1198. https://doi.org/10.1073/pnas.1119675109.

2. Cockerham, C.C., and Zeng, Z.B. (1996). Design III with Marker Loci. Genetics *143*, 1437–1456. https://doi.org/10.1093/genetics/143.3.1437.

3. Cordell, H.J. (2002). Epistasis: what it means, what it doesn't mean, and statistical methods to detect it in humans. Hum Mol Genet *11*, 2463–2468. https://doi.org/10.1093/hmg/11.20.2463.
